# The Turkish Bath

**Published:** 1861-07

**Authors:** 


THE TURKISH BATH.—This bath is attracting much attention in England, and the probability is that it will meet with great favor in that country. It has lately been inaugurated, with due honors, in Australia. It will, no doubt, be soon introduced into the United States. From a brief article in the *London Medical Times and Gazette*, which we here extract, it appears that it has already been employed with signal benefit in at least one lunatic asylum, namely, that of the Cork District, in Ireland:—

"At the meeting of the Cork District Lunatic Asylum last week a very interesting report was read by Dr. Power, resident physician, on the working of the Turkish bath in the asylum. It was introduced on his recommendation, aided by the inspectors, Drs. Nugent and Hatibel, approved by the governors, and sanctioned by the Privy Council. The experiment has been eminently successful. Since the opening of the bath in January last 124 patients have been submitted to its action. Out of these 10 are discharged cured, while 52 are improved and improving. Sixty remain unchanged, being all cases of many years' standing. Dr. Power says: 'I cannot conclude this report without stating, as the result of my limited experience, that such a provision should constitute an appendage to every institution where many persons are congregated for a lengthened period within a confined space. It seems to me to possess a power of compensating in a degree for the absence of pure air and exercise; and it seems to me a Turkish bath should form a part of every union workhouse and government prison, and all similar establishments.'"

